# A Biological Inventory of Prophages in *A. baumannii* Genomes Reveal Distinct Distributions in Classes, Length, and Genomic Positions

**DOI:** 10.3389/fmicb.2020.579802

**Published:** 2020-12-03

**Authors:** Belinda Loh, Jiayuan Chen, Prasanth Manohar, Yunsong Yu, Xiaoting Hua, Sebastian Leptihn

**Affiliations:** ^1^Zhejiang University-University of Edinburgh (ZJU-UoE) Institute, Zhejiang University, Haining, China; ^2^Department of Infectious Diseases, Sir Run Run Shaw Hospital, Zhejiang University School of Medicine, Hangzhou, China; ^3^Key Laboratory of Microbial Technology and Bioinformatics of Zhejiang Province, Hangzhou, China; ^4^University of Edinburgh Medical School, Biomedical Sciences, College of Medicine & Veterinary Medicine, The University of Edinburgh, Edinburgh, United Kingdom

**Keywords:** bacteriophage, prophage, *A. baumannii*, horizontal gene transfer, evolution, viral classification, antimicrobial resistance genes, phage genomes

## Abstract

*Acinetobacter baumannii* is of major clinical importance as the bacterial pathogen often causes hospital acquired infections, further complicated by the high prevalence of antibiotic resistant strains. Aside from natural tolerance to certain antibiotic classes, resistance is often acquired by the exchange of genetic information via conjugation but also by the high natural competence exhibited by *A. baumannii*. In addition, bacteriophages are able to introduce resistance genes but also toxins and virulence factors via phage mediated transduction. In this work, we analyzed the complete genomes of 177 *A. baumannii* strains for the occurrence of prophages, and analyzed their taxonomy, size and positions of insertion. Among all the prophages that were detected, *Siphoviridae* and *Myoviridae* were the two most commonly found families, while the average genome size was determined to be approximately 4 Mbp. Our data shows the wide variation in the number of prophages in *A. baumannii* genomes and the prevalence of certain prophages within strains that are most “successful” or potentially beneficial to the host. Our study also revealed that only two specific sites of insertion within the genome of the host bacterium are being used, with few exceptions only. Lastly, we analyzed the existence of genes that are encoded in the prophages, which may confer antimicrobial resistance (AMR). Several phages carry AMR genes, including OXA-23 and NDM-1, illustrating the importance of lysogenic phages in the acquisition of resistance genes.

## Introduction

The opportunistic pathogen *Acinetobacter baumannii* is the causative agent for bloodstream infections, meningitis and urinary tract infections, and is responsible for 2–10% of all Gram-negative hospital-acquired infections (Joly-Guillou, 2005). Such infections include ventilator-associated pneumonia and bacteremia with a mortality rate of 35–52% (Dijkshoorn et al., 2007; Kempf et al., 2013; Antunes et al., 2014). As a multitude of strains cause nosocomial infections, *A. baumannii* has become an important pathogen in hospital care and is of global concern. Many clinical isolates have acquired genes coding for virulence factors, such as toxins or efflux pumps, through various genetic uptake mechanisms (Morris et al., 2019). While *A. baumannii* easily acquires genetic material by conjugation, natural transformation is also widespread as many strains are highly naturally competent (Hu et al., 2019). Such mechanisms ultimately give rise to an increasing number of strains that display high levels of antimicrobial resistance (AMR), against which antibiotics show little or no effect. Genetic information for AMR genes is often embedded in genetic elements such as transposons or plasmids (Partridge et al., 2018; Rozwandowicz et al., 2018). In addition, bacteriophages (or phages) are able to transfer non-viral genetic information through a process called transduction, which can include genes coding for toxins or antimicrobial resistance (Wagner and Waldor, 2002; Derbise et al., 2007; Wachino et al., 2019). Therefore, phages play an important role in the development of AMR.

Regardless of their morphology or infection mechanism, phages can be divided into two types based on their life cycle: Lytic phages and lysogenic phages (sometimes also called temperate). Both eventually kill the host cell by lysis, employing various enzymes that create holes in the membrane and disintegrate the bacterial cell envelope, to allow the release of phage progeny. Few exceptions exist, such as the filamentous phages that are assembled in the membrane and secreted from the host while the bacterium continues to grow and divide (Loh et al., 2017, 2019; Kuhn and Leptihn, 2019). Nonetheless, phages that destroy the host by lysis upon completion of their life cycle, either start their viral replication immediately after entry (lytic phages) or integrate their genome into that of the host first (lysogenic). Lysogenic phages can remain “dormant” without replicating their genome or initiating phage coat protein synthesis. This way, lysogenic phages are being inherited by daughter cells, and might only replicate to form phage particles after many generations. The trigger for phage replication and synthesis is usually a stress signal produced by the host, such as a SOS response after DNA damage (Howard-Varona et al., 2017). However, if the host resides under favorable conditions, lysogenic phages continue their passive co-existence as so-called prophages embedded inside the DNA of the host.

Prophages are a major source of new genes for bacteria, occupying up to 20% of bacterial chromosomes and therefore may provide new functions to its host (Brüssow et al., 2004; Brüssow, 2007; Cortez et al., 2009; Fortier and Sekulovic, 2013; Wang and Wood, 2016). These functions include virulence factors and drug resistance mechanisms which include extracellular toxins and effector proteins involved in adhesion factors, enzymes, super antigens and invasion (Tinsley and Khan, 2006; Wang and Wood, 2016; Argov et al., 2017; Fortier, 2017). In some cases, the acquisition of virulence genes allows non-virulent bacteria to become a virulent pathogen. The most prominent example is that of the CTXΦ cholera toxin, encoded by a filamentous phage, making *Vibrio cholerae* the clinical pathogen that poses a substantial socio-economic burden on developing countries with poor hygiene due to frequent cholera outbreaks (Davis and Waldor, 2003). Another example is the Shiga toxin-encoding prophages found in highly virulent *Escherichia coli* strains, causing food-borne infections across the world (Gamage et al., 2004; Tozzoli et al., 2014). As part of the bacteriophage life cycle, prophages of lytic phages are a double edged sword; while they provide the advantage of increasing chances of survival in challenging environments, they could also lead to the killing of the host through the release of progeny at the end of the phage life cycle. As our relationship with microorganisms is a complex and vital one, from the important role of gut microbiota to the increase in mortality due to virulent microorganisms, understanding bacterial genomes is crucial. Yet in order to obtain detailed insight, we also need to be able to identify viral genes and to comprehend the impact of these genes on its host. As part of the bacterial genome, prophages are subjected to the general effects of mutation, recombination and deletion events. For some phages it has been clearly established that prophage genes have an influence on the host, such as motility or biofilm formation, both important aspects with regards to virulence. However for many other prophages, it is less well understood. Previous work on *A. baumannii* prophages have identified putative virulence factors and antibiotic resistance genes in host genomes deposited on GenBank (Costa et al., 2018; López-Leal et al., 2020). The work presented here analyses clinical *A. baumannii* strain genomes in search of possible prophages. We describe the identification of active prophages, analyzed their taxonomy, size and detail the regions in the bacterial genomes where these prophages have been found. Our data reveal a wide variation in the number of prophages in *A. baumannii* genomes and the prevalence of certain prophages within strains. From an evolutionary perspective, these might represent the “most successful” phages, or the ones that bring a benefit to the host. Our data analysis also allowed us to identify two major sites of insertion within the genome of the host bacterium, with most phage genomes inserting in these two regions, while only a few exceptions are being observed. In addition, our study indicates two distinct genome size distributions of prophages, as we observe a bimodal distribution when analyzing all prophage genomes. Furthermore, we describe genes coding for virulence factors, in particular for antimicrobial resistance in the prophages.

## Materials and Methods

*A. baumannii* genomes: Complete genome sequences of *A. baumannii* only were selected for this study. Detailed information of each *A. baumannii* strain used in this study is disclosed in supplementary material (Supplementary Table S1). For the characterization of genome lengths of *A. baumannii*, the following values were obtained: mean, median, mode, the smallest and the largest genome. The distribution of genome lengths was plotted using the geom_density function provided in the ggplot2 package in R (Wickham, 2016).

Alignment of *A. baumannii* genomes: All strains were aligned so that their starting position is identical, with the gene *dnaA* defined as the start. BLAST Scoring Parameters provided by NCBI (Gertz et al., 2005; available at https://blast.ncbi.nlm.nih.gov/Blast.cgi) was used to blast the locations of *dnaA* and the adjustment of genome sequences was achieved by SnapGene software (from Insightful Science; available at snapgene.com).

Identification of Prophage Genes: The tool used to identify prophages in *A. baumannii* genomes was Prophage Hunter (Song et al., 2019; available at https://pro-hunter.bgi.com/). Here, we obtained data on the start, end, length, score, category, and the name of the closest phage. Phaster (Arndt et al., 2016; available at http://phaster.ca/) was used to further confirm some conflicting results. According to the algorithm created by the authors of Prophage Hunter, an “active” prophage is defined by a score close to 1, while the probability decreases the lower the score gets. This means that an active prophage region received a scoring of higher that 0.8 while 0.5–0.8 is defined as “ambiguous,” and a score lower than 0.5 as “inactive.”

Prophage number analysis: Calculations of the mean, median, and mode on the prophage number (total, only active, and only ambiguous) were performed after removing the overlaps of the same prophage in the same strain. The 10 strains with the fewest and with the largest numbers of total, only active, and only ambiguous prophages were selected to show the 2 extremes, while the density plot achieved through geom_density function provided by ggplot2 package in R were conducted to describe the general distribution. The boxplot produced by R reflected the relationship between total, only active, and only ambiguous prophage number and *A. baumannii* genome length.

Phylogenetic analysis of host strains: Prokka v1.13 (Seemann, 2014) was used to generate the gff files for the genome sequences of 177 *A. baumannii* strains. The core genome alignment was constructed with Roary v3.12.0 (Page et al., 2015). A maximum-likelihood phylogenetic tree was created using FastTree v2.1.10 (Price et al., 2010). The tree was annotated and visualized with ggtree.

Prophage classification and phylogeny analysis: Prophage classification presented was provided by the program Prophage Hunter which was based on the NCBI’s database. Different orders and families were taken into consideration. The prophage number in different families and their proportions were revealed by histograms and pie charts generated in Microsoft Excel, respectively. Different bacterial hosts were referred to in the calculation of the number of prophages and the number of phage species they had. Based on the species of prophages, 10 most common ones for total, only active, and only ambiguous were selected, with a heatmap which was completed through geom_tile provided by ggplot2 package in R showing their number with the activity value in different strains. The phylogeny of phages was mapped according to their sequences. The alignment of the phage sequences were performed using Multiple Alignment using Fast Fourier Transform (MAFFT) (Katoh and Standley, 2013) with default options. Maximum-likelihood phylogenetic trees were created using FastTree v2.1.10. The tree was annotated and visualized using ggtree.

Prophage location analysis: The positions of all prophages were first showed in a stacked bar chart in Microsoft Excel. Considering the overlaps of different prophages in the same strain, all the prophage starts and ends were mapped in a density plot created by ggplot2 geom_density and aes functions in R. The stacked bar charts of different prophage species were used to estimate their preference of insertion which were then summarized in tables.

Prophage length analysis: The use of ggplot2 geom_density function in R facilitated the creation of density plots of prophage length. With the help of aes function, the mapping of prophage categories (active and ambiguous) and families were achieved in the density plots.

Identification of virulence factors and antibiotic resistance genes: No program is currently available that allows the search of virulence genes which are embedded in prophage sequences within bacterial genomes. Thus, we identified prophages first followed by manually correlating genomic positions of virulence genes with those that were also identified to belong to prophage genes.

Mapping of prophage-encoded antimicrobial resistance genes (ARG): To search for the specific virulence genes we identified (above), we first downloaded all available 4,128 *A. baumannii* Illumina sequencing reads from the Sequence Read Archive (SRA) with the cut-off date for deposited sequences on 2019/11/17. The raw Illumina sequencing reads were mapped against the ARG prophage sequences employing BWA-MEM v0.7.17 (80% coverage cutoff) (Li, 2013).

## Results

### *In silico* Discovery of Prophages in 177 *A. baumannii* Genomes Identifies 1,156 Prophage Sequences

**FIGURE 1:**
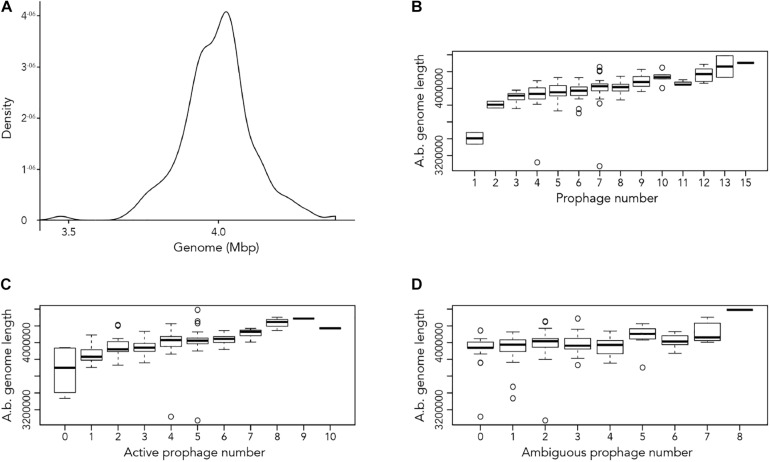
Length of *A. baumannii* genomes and its correlation with number of prophages present. **(A)** Density graph of 177 genome sequences of *A. baumannii* strains, indicating the distribution of the lengths of genome sequences analyzed. **(B–D)** Correlation between *A. baumannii* genome length and number of **(B)** all prophages, **(C)** active prophages, and **(D)** ambiguous prophages identified in genomes.

Our first aim was to analyze how frequently prophages occur in the genomes of *A. baumannii* strains. We randomly chose 177 genome sequences of *A. baumannii* strains, many of them clinical isolates. For the subsequent analyses, we aligned all sequences so that their starting positions are identical. To this end, we defined the gene *dnaA* as the start, which codes for a replication initiation factor that facilitates DNA replication in bacteria. From the sequence alignments, we observed a large variation in genome sizes. The average length of the genomes was 3,981,579 bp with a median value of 4,001,318 bp; the smallest genome had a length of 3,072,399 bp (22.9% shorter than average), and the largest genome displayed a size of 4,389,990 bp (10.25% larger than average) (Figure 1A).

**FIGURE 2:**
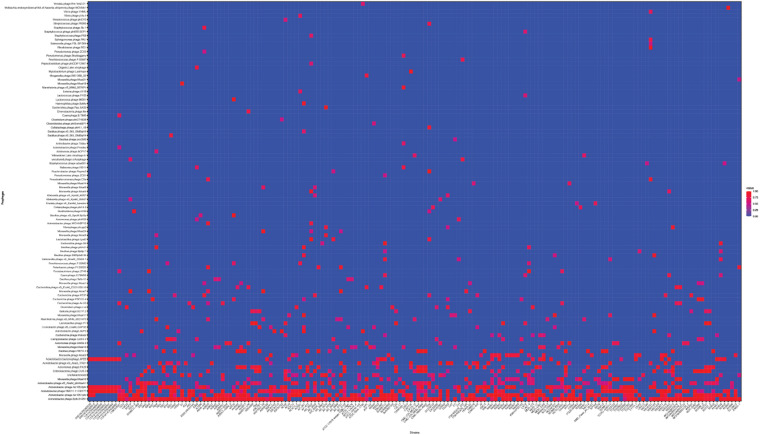
The prevalence of prophages analyzed. Heat map of prophages found in all *A. baumannii* strains analyzed. Prophages (*y*-axis) are plotted against each *A. baumannii* strain (*x*-axis). Red squares indicate the presence of the indicated prophage. Blue squares indicate the lack thereof. Please refer to the PDF of the figure and use the zoom function to identify names of strains and phages.

Next, we used the online platform Prophage Hunter (Song et al., 2019) to identify active and ambiguous prophage genomes. The algorithm provides an output value for each prophage identified, which allows the researcher to establish whether a sequence contains an “active” or an ambiguous prophage. While “active” prophages exhibit the complete genomic sequence of a prophage, and are therefore likely to allow the production of phage particles, ambiguous prophage sequences are truncated, mutated or otherwise incomplete, and unlikely to be able to form infectious phages. Among the 177 *A. baumannii* genomes analyzed, we identified 1,156 prophages, with 459 of them being defined as “ambiguous” while the remaining 697 sequences were labeled as “active” according to the program (Supplementary Table S2). To determine the prevalence of prophages in the *A. baumannii* genomes, we analyzed the number of prophages per genome. Using a heatmap to illustrate our results, we found that while some prophages are rarely found, others are quite common in the genomes of *A. baumannii* isolates (Figure 2). One phage that was only found once, for example, is a prophage with high sequence similarity to the *Yersinia* Podovirus fHe-Yen3-01, while the *Acinetobacter* phage Bphi-B1251, a Siphovirus, has been found in 79.1% (140 in 177) of all analyzed *A. baumannii* genomes. Such high prevalence observed by prophages such as Bphi-B1251 could indicate high infectivity and wide host range of the active phage particle. Table 1 shows 10 strains with the fewest and with the largest numbers of prophages identified (Table 1). We found that the average prophage number in an *A. baumannii* genome is 6.53, with some bacterial genomes containing only one (*n* = 2) prophage sequence, such as in case of the *A. baumannii* strains DS002 and VB1190. In contrast to these, other strains have been found to contain as many as 10 prophage sequences, such as in strain 9201 (*n* = 1), that were labeled “active” by Prophage Hunter; additionally this strain contains two prophage sequences that were defined as ambiguous. The highest prophage number was found in the strain AF-401 which contains 15 prophages, however, only 8 were defined as active. Our results show that prophage sequences are relatively common and that most *A. baumannii* strains show a median of seven and a mode of eight prophages per genome. Additional genome analysis of the clinical isolates illustrates a possible relationship between prophages and host strains (Supplementary Figure S1). Prophages, such as *Bacillus* phage PfEFR 4 and *Enterobacteria* phage CUS 3 were observed more frequently in strains whose genomes are in the same clade or are closely related, indicating a narrow host range.

**TABLE 1:** *A. baumannii* stains with the highest and the fewest number of prophages identified.

Highest number of prophages identified		Fewest number of prophages identified	
Strain	Prophage number	Strain	Prophage number
AbPK1	11	DS002	1
AR_0056	11	VB1190	1
9201	12	CA-17	2
10042	12	E47	2
AR_0101	12	11A14CRGN003	3
DU202	12	11A1213CRGN008	3
VB35435	12	11A1213CRGN055	3
11W359501	13	11A1213CRGN064	3
AB030	13	11A1314CRGN088	3
AF-401	15	11A1314CRGN089	3

We next correlated host genome length with number of prophages identified to determine if there is a relationship between the two variables. Perhaps unsurprisingly, the length of *A. baumannii* genomes increased as more prophages are identified, disregarding whether the prophage genomes are “active” or “ambiguous” (Figure 1B). However, when prophages are classified, the correlation between host genome length and number of prophage genomes identified were less distinct (Figures 1C,D).

### *Siphoviridae* and *Myoviridae* Are the Two Most Commonly Found Classes of Prophages in *A. baumannii* Genomes

**FIGURE 3:**
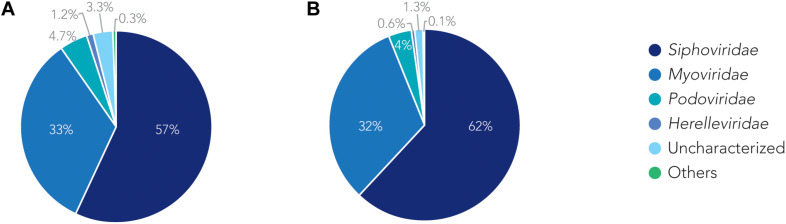
The families of prophages found. Pie charts of prophages identified showing the percentage make up of each family. **(A)** Classification for all prophages. **(B)** Classification of active prophages only.

We analyzed the relationship of all prophages we identified and created a phylogenetic tree (Supplementary Figure S2). Phage phylogeny is very complex. While bacteria share many common genes, microbial viruses are less related to each other creating large phylogenetic distances. The phylogenetic analysis shows that in some instances the phylogenetic clustering does not necessarily result in grouping of the phages according to their classes. This is, however, not surprising as phages often display diversity by “mosaicity of their genomes” (Dion et al., 2020). After identifying all prophage sequences in the genomes of the 177 *A. baumannii* isolates, we set out to analyze the most prevalent classes of phages present. Our analysis of all prophage sequences (*n* = 1,156) revealed that the majority of them, ∼57% (*n* = 660) of the prophages, belong to the *Siphoviridae* group (Figure 3A). The *Siphoviridae* is a class of head-and-tail phages, with the best known representative being phage lambda, that exhibit long, non-contractile but comparably flexible tail structures (Nobrega et al., 2018). The second most commonly found prophages are *Myoviridae*, with a percentage of ∼33% (*n* = 385) (Figure 3A). With the best-known Myovirus, the *E. coli* phage T4, these phages have a stiff, contractible tail that allows the active penetration of the bacterial host envelope (Hu et al., 2015). Together, these two phage classes make up 90% of all prophage genomes. The third most common class, albeit only 4.7% (*n* = 55) of all prophage genomes, belongs to *Podoviridae*. The best known Podovirus is probably T7, which has a short, stubby tail and internal core proteins that get ejected for the formation of a DNA-translocating channel across the bacterial cell envelope (Guo et al., 2014; Lupo et al., 2015; Leptihn et al., 2016). Prophages that could not be conclusively classified to a viral group accounted for 3.3% (*n* = 38). *Siphoviridae*, *Myoviridae* and *Podoviridae* all belong to the order *Caudovirales*, phages that exhibit a head-and-tail structure. Within the two phage classes we found several phages that were most successful, i.e., most common. Examples are the *A. baumannii* phages Bphi-B1251 and YMC11/11/R3177, which both belong to the *Siphoviridae* (Table 2A). The most common Myovirus was Ab105-1phi. Not only are these the most common prophages found, they are also the most common active prophages identified (Table 2B). In addition, the distribution of the classes was similar if only active prophages were analyzed. Here, 62% (432/697) belonged to the *Siphoviridae* and 32% (223/697) to the *Myoviridae* (Figure 3B).

**TABLE 2:** The most common prophages identified in *A. baumannii* stains.

A	10 the most common prophages (total)		B	10 the most common prophages (active)	
	Phages	Number found		Phages	Number found
	*Acinetobacter* phage Bphi-B1251	228		*Acinetobacter* phage Bphi-B1251	177
	*Acinetobacter* phage Ab105-1phi	143		*Acinetobacter* phage Ab105-1phi	111
	*Acinetobacter* phage YMC11/11/R3177	118		*Acinetobacter* phage YMC11/11/R3177	78
	*Acinetobacter* phage Ab105-2phi	95		*Acinetobacter* phage Ab105-2phi	65
	*Acinetobacter* phage vB_AbaM_phiAbaA1	56		*Aeromonas* phage PX29	31
	*Moraxella* phage Mcat16	42		*Enterobacteria* phage CUS-3	25
	Uncharacterized	38		*Acinetobacter* bacteriophage AP22	23
	*Enterobacteria* phage CUS-3	35		*Bacillus* phage PfEFR-4	20
	*Aeromonas* phage PX29	34		*Acinetobacter* phage vB_AbaS_TRS1	19
	*Acinetobacter* phage vB_AbaS_TRS1	33		*Moraxella* phage Mcat3	16

### The Genomic Position of Prophages Shows Two Main Locations for Genome Integration

**FIGURE 4:**
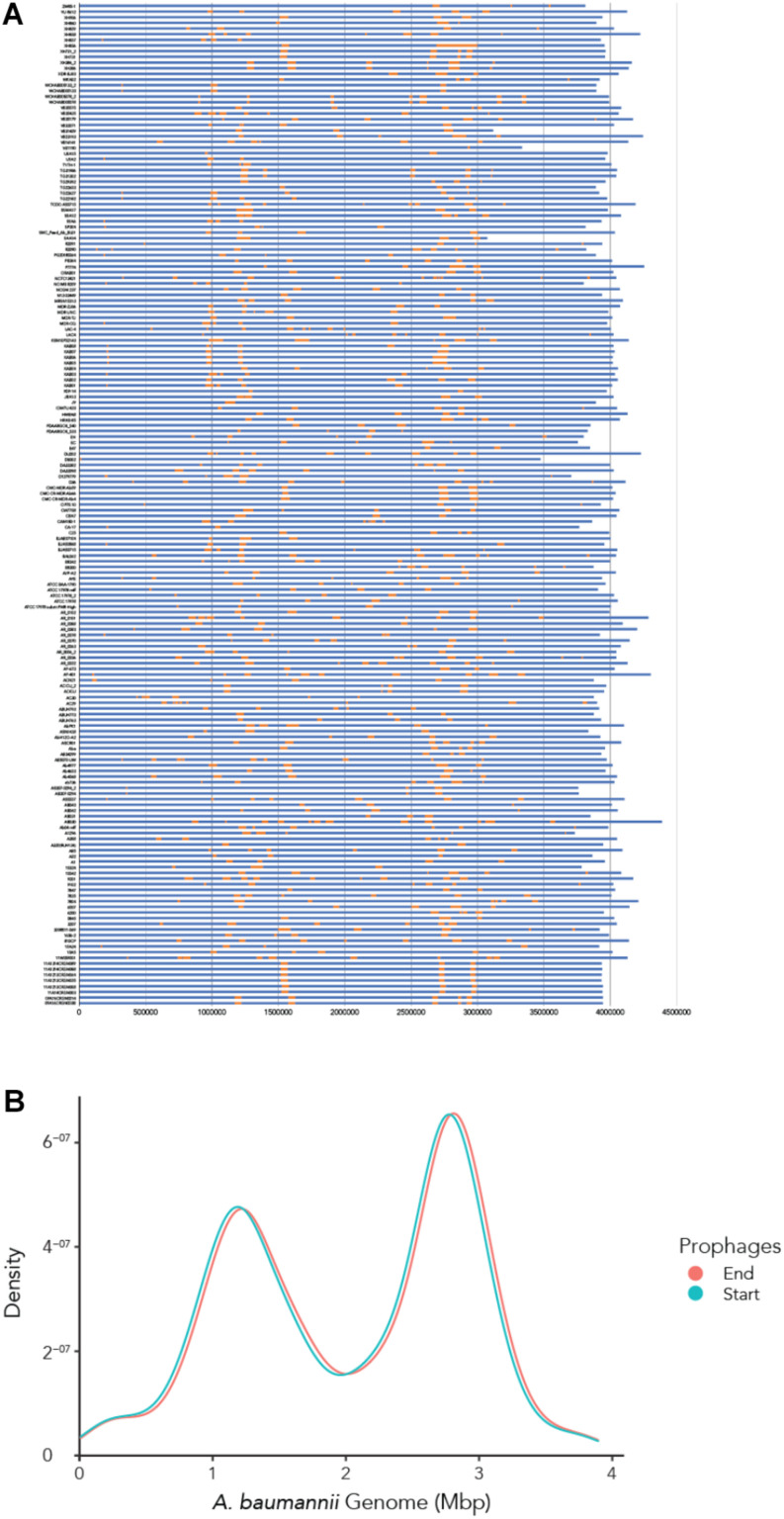
The location of prophages found in each *A. baumannii* genome. **(A)** Stacked bar chart of each bacterial strain (*y*-axis). Yellow segments indicate prophage sequences identified on the genome (in blue). **(B)** Density graph compiled from the stacked bar chart. Please refer to the PDF of the figure and use the zoom function to identify names of strains and phages.

**FIGURE 5:**
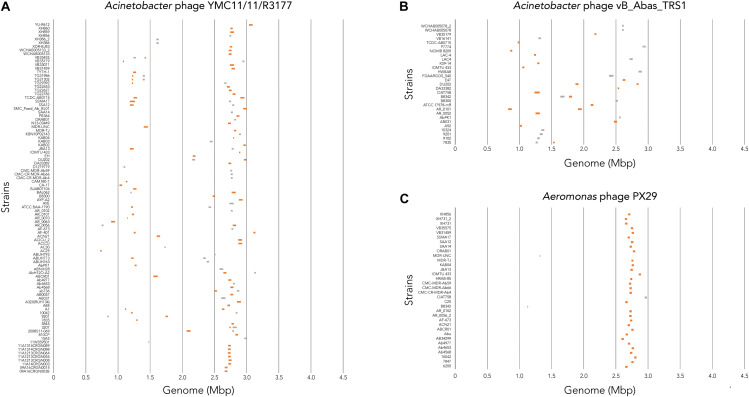
Location of prophage genome insertion differs between phages. **(A)** Comparison of phage location for *Acinetobacter* phage YMC11/11/R3177. **(B)** Comparison of insertion locations for *Acinetobacter* phage vB_Abas_TRS1. **(C)** Comparison of prophage insertion sites for *Aeromonas* phage PX29. Boxes in orange indicate active prophages identified. Gray boxes indicate ambiguous prophage sequences. Please refer to the PDF of the figure and use the zoom function to identify names of strains and phages.

To determine where all prophages -regardless of their class- are found in the bacterial genome, or if they are possibly distributed at random within the host DNA, we visualized the position of the prophages in all 177 genomes (Figure 4A). We then plotted all prophage positions found in all genomes against the position in the bacterial host genome sequence. Surprisingly, we observed a bimodal distribution, with two clear peaks in the position of prophages (Figure 4B), indicating that there are two main sites of attachment for prophages and their genomic insertion. While this reflects the situation for all prophages, we then analyzed the position of several individual prophages within the bacterial genome. First, we assessed one of the most commonly found phages YMC11/11/R3177. The position of this phage reflects the overall distribution of all phages in the analyzed genomes, with two main areas of insertion. However, in some cases the position is outside the main area of insertion, possibly due to recombination of the bacterial genome (Figure 5A). The second phage we analyzed was phage vB_AbauS_TRS1. Here, the distribution of the phage within the bacterial genome seems to be more random as compared to the overall distribution (Figure 5B). In case of the *Aeromonas* phage PX29, insertion seems to be very “strict,” i.e., only observed in one location within the genome (Figure 5C). The observations made when analyzing the prophage positions show that the insertion of phages could be described as “directed,” and less random, indicating that attachment sites, if they are required, are found more commonly in certain positions of the bacterial genome.

### The Sequence Length of Prophages Reveals Distinct Groups

**FIGURE 6:**
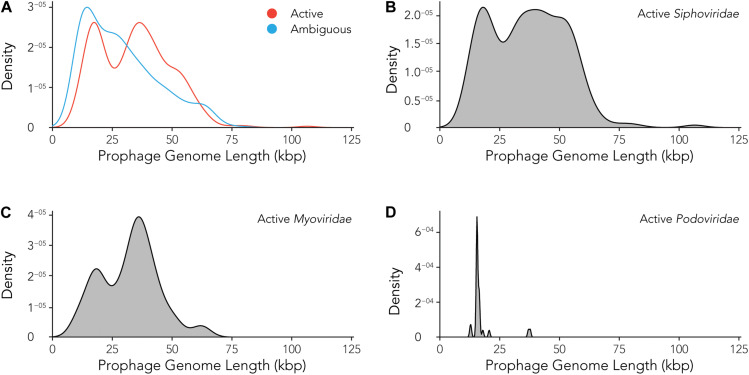
Active prophages categorized by prophage lengths. **(A)** Comparison of prophage lengths between active and ambiguous prophages. **(B–D)** Distribution of prophage length for active *Siphoviridae* **(B)**, *Myoviridae* **(C)**, and *Podoviridae* **(D)**.

Using the data provided by the program Prophage Hunter, we were interested in evaluating the size distribution of prophage genomes. Therefore, we plotted sizes against the frequency of prophages present in the bacterial genomes and calculated average prophage genome sizes. In the case of ambiguous prophages, a main population at 15 kb became visible followed by a minor peak of substantial size at approximately 60 kb, leaving the average and median length of ambiguous prophage genomes at 29.2 and 25.8 kb, respectively (Figure 6A and Table 3). In contrast to this, two main peaks were observed when analyzing only active prophages. Here, one peak is observed at around 17 kb while the other at around 36 kb was observed (Figure 6A). The average genome length of active prophages is 34 kb (Table 3). As these peaks include all phage categories, we re- analyzed the genomic length of the active prophages according to their classes: *Siphoviridae*, *Myoviridae* and *Podoviridae*, which together constitutes almost 95% of all prophages (see Figure 3A). When analyzing the length of all *Siphoviridae* sequences, we observed two main populations, one sharp peak at around 20 kb and one broad peak with a shoulder containing larger sequences from 18 to 56 kb (Figure 6B). The average prophage length of *Siphoviridae* is 36.7 kb (Table 3). *Myoviridae* sequences similarly exhibited two sharp peaks (17 and 36 kb), with a third minor one of around 60 kb (Figure 6C). The average prophage genome size of active *Myoviridae* is 32.4 kb (Table 3). The *Podoviridae* showed several minor peaks with a large sharp peak at about 12 kb and the average prophage genome length is calculated to be 17.4 kb (Figure 6D and Table 3). The results of these analyses show that there are distinct distributions of bacteriophage genome sizes. Two clearly separated groups of prophages can be observed just based on size, in the case of *Myoviridae*. In the case of *Siphoviridae*, we saw a less defined area with possibly multiple species within the broad distribution.

**TABLE 3:** Prophage genome lengths for active and ambiguous prophages, among the three major families in the order of *Caudovirales*.

		All prophages	Active prophages	Ambiguous prophages
All prophages	Average	32211.62	34182.66	29218.56
	Median	31,296	35,074	25,759
*Siphoviridae*	Average	34586.75	36668.1	30625.76
	Median	33,018	36562.5	27,679
*Myoviridae*	Average	32148.62	32351.98	31870.4
	Median	34,249	35,075	30,792
*Podoviridae*	Average	19422.25	17357.86	21563.11
	Median	15,498	15,498	14,963

### Prophage Encoded Antibiotic Resistance Genes

As prophages are able to encode genes that might allow its host to become more virulent and therefore more evolutionary successful, we aimed to analyze prophage-encoded virulence factors. However, in contrast to e.g., *E. coli*, a databank for *A. baumannii* virulence factors currently does not exist. We therefore searched for prophage sequences that contain genes that contribute to antibiotic resistance. Table 4 lists the start and end of the genes that are encoded within a respective prophage. Among others, we found AMR genes for OXA-23 and NDM-1. OXA-23 is the most widespread carbapenem resistance gene globally (Hamidian and Nigro, 2019). NDM-1 encodes a carbapenemase, a beta-lactamase enzyme with a broad substrate specificity capable of hydrolyzing penicillins, carbapenems, cephalosporins, and monobactams. Other beta-lactamase genes were *bla*_*ADC*__–__5_, *bla*_*OXA*__–__67_, *bla*_*OXA*__–__115_, and *bla*_*TEM*__–__12_. In addition, we were able to identify genes coding for N-Acetyltransferases(aac(3)-I, *aac(3)-Id*, *aacA16)*, Aminoglycoside phosphotransferases (*aph(3′)-Ia*, *aph(3′)-VI*, *aph(6)-Id*, *aph(3″)-Ib*), both groups mediating aminoglycoside resistance. Other genes that contribute to antibiotic resistance were sulfonamide resistance gene (*sul2*), and the macrolide-resistance conferring genes *msr(E)*, encoding an efflux pump, and *mph(E)*, coding for a macrolide-inactivating phosphotransferase.

**TABLE 4:** Antimicrobial resistance genes found in prophages embedded in *A. baumannii* stains.

Strain	AMR gene	Start of resistance gene	End of resistance gene	Phage name	Start of prophage sequence	End of prophage sequence
AB030	blaADC-5	3131953	3133104	*Acinetobacter* phage Bphi-B1251	3061121	3133273
AB5075-UW	blaOXA-23	562998	563819	*Escherichia* phage vB_EcoM_ECO1230-10	545886	581029
AbPK1	aac(3)-I	1359578	1360042	*Acinetobacter* phage Ab105-1phi	1357668	1404464
ABUH793	blaOXA-115	2017307	2018131	*Clostridium* phage phiCT453B	2016119	2031153
AC29	blaTEM-12	728807	729667	*Acinetobacter* phage YMC11/11/R3177	723894	746651
AC29	aph(3’)-Ia	732720	733535	*Acinetobacter* phage YMC11/11/R3177	723894	746651
ACN21	aph(3’)-VI	110687	111466	*Vibrio* phage pVa-4	98299	123387
ACN21	blaNDM-1	112744	113556	*Vibrio* phage pVa-4	98299	123387
ACN21	ble-MBL	113560	113925	*Vibrio* phage pVa-4	98299	123387
ACN21	ble-MBL	113560	113925	*Listeria* phage A118	113004	131863
AR_0056	sul2	3643229	3644044	*Moraxella* phage Mcat6	3631231	3645626
AR_0078	aph(3’)-Ia	1454389	1455204	*Bacillus* phage PfEFR-4	1448981	1463460
AR_0078	msr(E)	1456488	1457963	*Bacillus* phage PfEFR-4	1448981	1463460
AR_0078	mph(E)	1458019	1458903	*Bacillus* phage PfEFR-4	1448981	1463460
BJAB0715	blaOXA-23	1040633	1041454	*Pseudomonas* phage ZC01	1035624	1064722
DU202	blaOXA-23	1304225	1305046	*Escherichia* phage PBECO 4	1300481	1331113
DU202	aac(3)-Id	1307973	1308424	*Escherichia* phage PBECO 4	1300481	1331113
EC	blaOXA-67	2088567	2089391	*Bacillus* phage proCM3	2082124	2102020
LAC4	aph(6)-Id	3852495	3853331	*Lactococcus* phage P162	3843825	3855365
LAC4	aph(3″)-Ib	3853331	3854133	*Lactococcus* phage P162	3843825	3855365
MDR-UNC	aacA16	1317505	1318056	*Aeromonas* phage PX29	1315787	1325918
MDR-UNC	aac(3)-I	1321744	1322208	*Aeromonas* phage PX29	1315787	1325918
TCDC-AB0715	sul2	2557327	2558142	*Acinetobacter* phage vB_AbaM_phiAbaA1	2530565	2567304
XH858	blaOXA-23	1093489	1094310	N/A	1088870	1114410
XH859	blaOXA-23	1070735	1071556	*Acinetobacter* phage Bphi-B1251	1038026	1092046

Interestingly, one *A. baumannii* strain, ACN21, contains a prophage which encodes three antibiotic resistance genes [*aph(3’)-VI*, *bla*_*NDM*__–__1_, *ble*_*MBL*_]. The phage is most closely related to *Vibrio* phage pVa-4, a Myovirus that infects *V. alginolyticus* (Kim et al., 2019). In contrast to its relative, phage pVa-4 is a lytic phage was grouped to be part of the phiKZ-like phages (Phikzviruses), which are considered as “jumbo” phages. A second *A. baumannii* strain, AR_0078, contains a prophage sequence that shares a high degree of similarity to the *Bacillus* phage PfEFR-4, a Siphovirus with a prolate head, in contrast to its *E. coli* relative lambda (Geng et al., 2017). The prophage encodes three antimicrobial resistance genes that confer macrolide-resistance, *msr(E)*, encoding an efflux pump, and *mph(E)*, coding for a macrolide-inactivating phosphotransferase. The third gene encodes the Aminoglycoside phosphotransferases [*aph(3’)-Ia*], inactivating aminoglycoside antibiotics.

*A. baumannii* strain DU202 contains a prophage sequence that is related to the lytic *E. coli* myovirus PBCO 4 (Kim et al., 2013). Within the genome sequence, two antimicrobial resistance genes are encoded: *aac(3)-Id* codes for an N-Acetyltransferase mediating aminoglycoside resistance and OXA-23, which is encodes the most widespread resistance mechanism toward the β-lactamase inhibitor sulbactam. The gene was also embedded in the prophage sequences of two phages in the *A. baumannii* strain XH859; here, the *A. baumannii* phage Bphi-B1251 was found to be the most closely related phage, a lytic Podovirus, that was previously shown to be able to infect and lyse an OXA-23- harboring *A. baumannii* isolate from a septic patient (Jeon et al., 2012).

To determine how prevalent the prophage encoded antimicrobial resistance genes are in other genomes, we mapped all available 4,128 *A. baumannii* Illumina sequence reads that were accessible by 2019/11/17 on the Sequence Read Archive (SRA) to the AMR prophage sequences using an 80% cutoff for the coverage. We identified 174 *A. baumannii* genomes that contain prophage sequences, or about 4.2% of all available reads, not including the genomes we used for the initial analysis. Supplementary Figure S3 illustrates the prevalence of the prophage-encoded AMR genes (ARGs) and their respective prophages. Fairly “successful,” i.e., widely distributed, were two prophages: ABUH793 and AR_0056. ABUH793 is a close relative of the *Clostridium* phage phiCT453B, containing the resistance gene *bla*_*OXA–115*_. The second prophage sequence that was found often in *A. baumannii* genomes in comparison to other prophages encoding ARGs, was AR_0056, a relative of the *Moraxella* phage Mcat6, encoding the ARG *sul2*. While the prophage does not necessarily render the host antibiotic resistant as genetic regulators might be missing, prophages containing ARGs can present an evolutionary advantage for the host (Wendling et al., 2020).

Our finding demonstrates the importance of phages in the acquisition of antimicrobial resistance; the above described genes may confer the ability to grow in the presence of antibiotics when the bacterial host is infected by a phage that encodes not only the information for its own replication but also genes that inactivate or remove antibiotic compounds.

## Discussion

Our search for prophages in the genomes of *A. baumannii* strains revealed several interesting findings. One surprising observation was the positions of the prophages within the genome of the bacterial host. When analyzing the prophage positions one might expect that the insertion of phages would be less directed, and more random. However, we found that the majority of phages inserted into two locations as seen by a bimodal density plot with a sharp separation between the two peaks. Prophage genome integration can either be a site-specific recombination event at so-called *att* sites or occurs in a non-directed manner by transposition into random sites (Ramisetty and Sudhakari, 2019). Our data could indicate that the two areas in the genome contain most of the attachment sites for the majority of phages. A previously published analysis of *Salmonella* and *E. coli* genomes found a large number of distinctive phage integration loci; in the case of *Salmonella*, 24 loci were shared among 102 *Salmonella* phages, amounting to four phages statistically sharing one integration site. In case of *E. coli*, 58 distinctive integration loci were identified for 369 phages, with statistically 6.6 phages per site (Bobay et al., 2013). It might be reasonable to assume that *A. baumannii* contains similar numbers of attachment sites, although we have not analyzed potential sites in the genomes we investigated. However, regardless of whether a phage inserts via one of the various attachment sites or randomly via transposition, only two “hot spots” were observed in our study. In addition to the explanation that attachment sites might be more frequent in these two sections of the bacterial genome, prophage insertion into segments crucial for e.g., over-all gene regulation, or into household genes, would be an evolutionary disadvantage and might therefore be less commonly found.

Bacteriophages are classified into 12 families (ICTV, 2019). Pioneering work in taxonomy divided tailed phages into three classes based on the morphology of the phages; *Myoviridae* have long contractile tails, *Siphoviridae* have long non contractile tails, and *Podoviridae* have short tails. Recently, the International Committee on Taxonomy of Viruses (ICTV) expanded the order *Caudovirales*, describing tailed bacteriophages to include six additional families, i.e.,: *Ackermannviridae*, *Autographiviridae*, *Chaseviridae*, *Demerecviridae*, *Drexlerviridae*, and *Herelleviridae*, taking additional characteristics into consideration such as genome sequence, gene content, protein homology and the host (Adriaenssens et al., 2020). When analyzing the families of prophages in this population of *A. baumannii* strains, we observed a prevalence of *Siphoviridae* which constituted 57% of all identified prophages. Together with the next family of phages, *Myoviridae*, which consists 1/3 of all prophages, the two groups make up 90% of all prophages identified. Among the remaining 10%, the largest group belongs to *Podoviridae*. These ratios are very similar to the ones that have been reported in other studies, and also the ratio of the most commonly found phages in nature (Costa et al., 2018).

Interestingly, the ratio between ambiguous to active prophages in case of the ones that have been identified as *Siphoviridae*, 0.654, markedly differs from the ratio calculated for the prophages that belong to *Myoviridae*, 0.579. It is unlikely that *Siphoviridae* prophage sequences are less prone to mutations, as they should be occurring at random. However, a mechanism that would specifically “protect” *Siphoviridae* prophages might be the case if daughter cells, where mutations in the prophages occur, would have an evolutionary disadvantage. This would imply that prophages influence the host behavior positively, which has previously been shown in some cases (Bondy-Denomy and Davidson, 2014; Nanda et al., 2015; Loh et al., 2019). Could the most likely scenario be that the genomes of *Myoviridae* are possibly larger than those of the *Siphoviridae*, making them more prone to random mutations and deletions? However, the size comparisons of the genomic sequences of the prophages that we identified, does not support this possible explanation: The *Siphoviridae* sequences display distribution with one clear peak at around 20 kb followed by a fairly broad peak with a plateau and a shoulder toward larger genome sizes, ranging from 28 to 65 kb (Figure 6B). In contrast to this, the genomic size distributions of *Myoviridae* showed two peaks, one around 20 kb, the second around 42 kb (Figure 6C). The size estimations are corroborated by the findings of a previous study which estimated the genome sizes of *Siphoviridae* to be approximately 50 kb in average, with a broad distribution between 24 and 101 kb, while *Myoviridae* display smaller genomes or around 34 kb (Costa et al., 2018).

One question we could pose is why there is no broad distribution of prophage sizes, and why do we observe “peaks”? Can we conclude from this data that certain genome sizes are advantageous from an evolutionary standpoint? The arch-Myovirus might have had a certain size that proved to be sufficient for the successful persistence during the course of evolution. Only smaller increases or decreases of the genome allowed evolutionary success, and no gradual increase or decrease in genome size occurred. However, an evolutionary leap or jump might have happened at some point, which might have led to a major increase of genomic size, creating a new, second type of a Myovirus class which is represented in the second, larger peak. Starting from this size, again only smaller changes, decreases or increases with regards to the genomic size, may have occurred, preserving the sharp separation of each peak. It would be interesting to investigate if the smaller Myovirus display a prolate head as does T4. The increase volume of this geometry allows the packing of a larger genome, which might explain the possible separation in two sizes. To test this hypothesis, smaller Myoviruses should have non-prolate heads. Viral classification is a complex topic. Possibly the genome sizes might help to contribute to classifying of microbial viruses in the future.

While Prophage Hunter extracts prophage genomes from bacterial genomes, the platform is a web-based tool that also distinguishes between “active” and “ambiguous” prophage genomes (Song et al., 2019). The developers of Prophage Hunter have used experimental data and conducted induction experiments with mitomycin C, to validate the program’s output, showing its ability to hunt for “active,” inducible prophages. Yet, conclusions should not be hastily drawn to assume that all “active” prophages can definitively excise from the host genome to commence the bacteriophage lytic life cycle; false positives may still exist. In this regard, induction experiments should be conducted to confirm that “active” prophages can indeed produce active particles.

Prophages are an important source for acquiring new genetic information, including antibiotic resistance genes, for their bacterial host. Phage-mediated transfer of genes from donor to recipient cells, also called transduction, has been shown to be instrumental in the spread of AMR genes both *in vitro* and *in vivo* (Haaber et al., 2016). In our study, we also investigated AMR genes that are embedded in prophage sequences. Previous studies on prophage diversity in *A. baumannii* had found AMR genes (also called: ARGs) in many prophages that were analyzed (Costa et al., 2018; López-Leal et al., 2020). Yet despite this, it remains to be shown whether prophages confer antimicrobial resistance to its host *A. baumannii*. Our observation illustrates that phages might represent important contributors in the process of AMR acquisition. However, it remains to be said that we found less than 5% of a publicly available, deposited sequence reads to contain the prophage-encoded ARGs we initially identified, arguing that phage transduction is possibly not the prevalent mode of AMR acquisition but is second to other mechanisms such as plasmid uptake via conjugation. Interestingly, despite viruses in general showing highly condensed genomes trying to pack essential information in small volumes, bacterial viruses seem to have co-evolved with their hosts and carry genes that are not directly required for the virus but are beneficial to the host and thus also to the prophage.

## Conclusion

Our study attempts to take an inventory of prophages in the important nosocomial pathogen *A. baumannii*. We have analyzed the phylogeny of the prophages, their position in the host genome and characterized their lengths, identifying “successful,” i.e., widely distributed phages, and the dominant families, *Myoviridae* and *Siphoviridae*.

Several prophage sequences contained genes coding for antimicrobial resistance genes. By mapping these genes in all deposited illumina *A. baummannii* sequence reads, we found that less than 5% of all available host sequences contain such prophage-embedded genes, indicating that transduction may not be the major contributor to the emergence of antimicrobial resistance.

## Data Availability Statement

The raw data supporting the conclusions of this article will be made available by the authors, without undue reservation, to any qualified researcher.

## Author Contributions

SL and XH devised this study. JC, BL, XH, and SL performed the work. BL created the figures. BL, PM, XH, YY, and SL wrote the manuscript. All authors contributed to the article and approved the submitted version.

## Conflict of Interest

The authors declare that the research was conducted in the absence of any commercial or financial relationships that could be construed as a potential conflict of interest.

## Supplementary Material

Supplementary Figure 1 *In silico* detection and distribution of prophage sequences in *A. baumannii* strains. Unrooted core phylogenetic tree of 177 *A. baumannii* genomes from clinical isolates. Presence of prophage sequences are indicated by red squares. Green squares indicate the lack thereof. Please refer to the PDF of the figure and use the zoom function to identify names of strains and phages.

Supplementary Figure 2 Phylogeny of prophages found in *A. baumannii* genomes.

Supplementary Figure 3 Prevalence of prophages carrying AMR genes in *A. baumannii* strains.

Supplementary Table 1 Metadata of all *A. baumannii* genomes used in this study.

Supplementary Table 2 Details of detected active and ambiguous prophage in all 177 *A. baumannii* strains analyzed in this study.
