# Supplementary figures and images for: A Biological Inventory of Prophages in A. baumannii Genomes Reveal Distinct Distributions in Classes, Length, and Genomic Positions
Source: Front Microbiol. 2020 Dec 3;11:579802. doi: 10.3389/fmicb.2020.579802 (PMC7744312; doi:10.3389/fmicb.2020.579802)

## Supplementary Figure S1

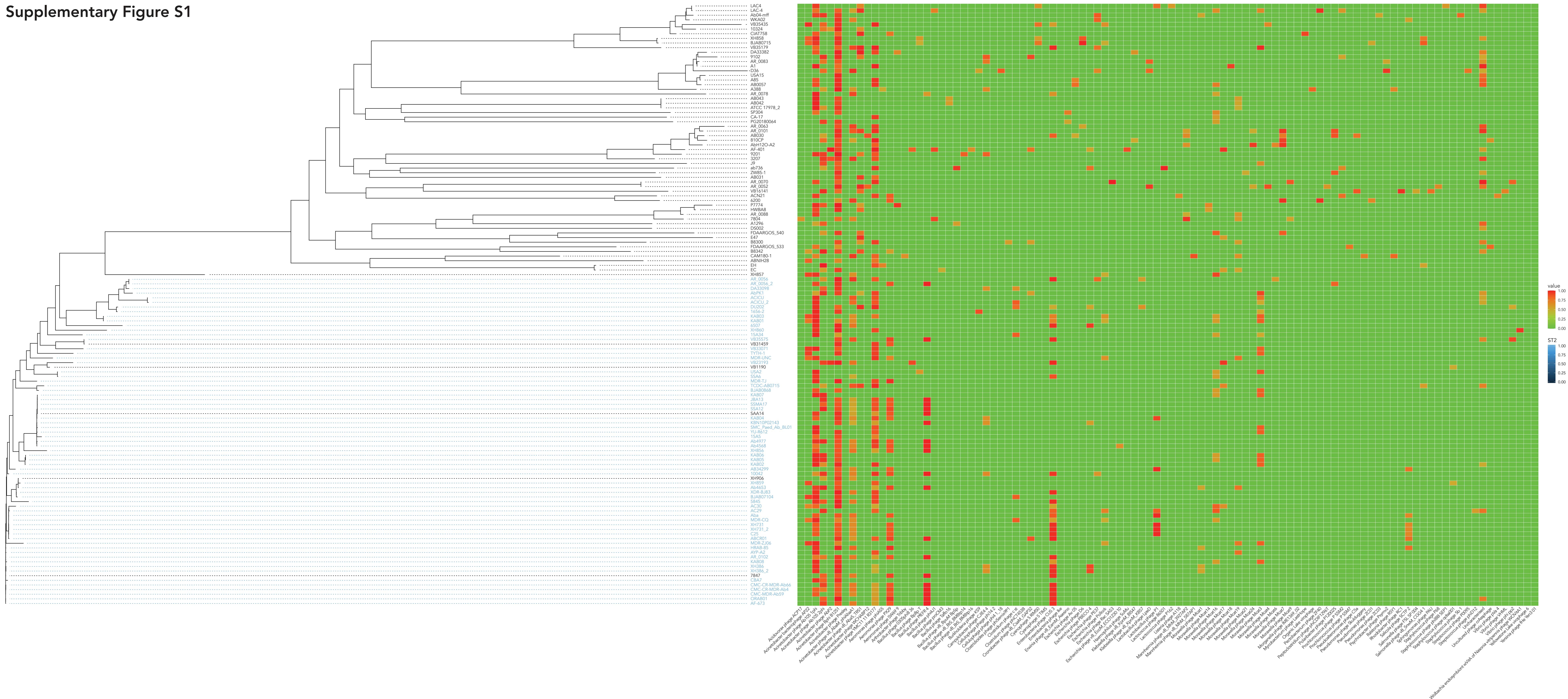

Supplement: Supplementary Figure 1 — In silico detection and distribution of prophage sequences in A. baumannii strains. Unrooted core phylogenetic tree of 177 A. baumannii genomes from clinical isolates. Presence of prophage sequences are indicated by red squares. Green squares indicate the lack thereof. Please refer to the PDF of the figure and use the zoom function to identify names of strains and phages. [file Image_1.pdf]

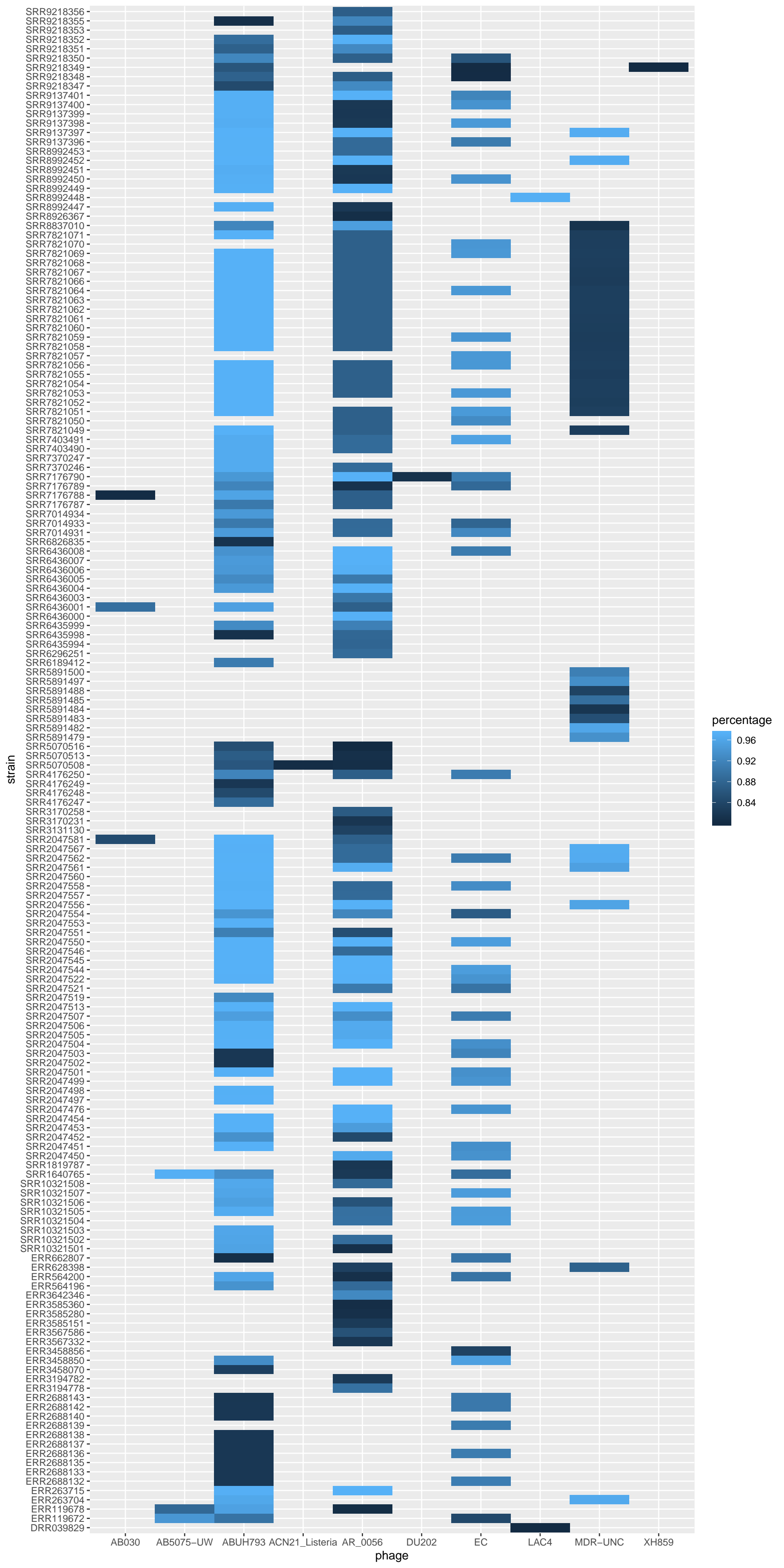

Supplement: Supplementary Figure 3 — Prevalence of prophages carrying AMR genes in A. baumannii strains. [file Image_3.pdf]
