# Supplementary material for: A Biological Inventory of Prophages in A. baumannii Genomes Reveal Distinct Distributions in Classes, Length, and Genomic Positions
Source: Front Microbiol. 2020 Dec 3;11:579802. doi: 10.3389/fmicb.2020.579802 (PMC7744312; doi:10.3389/fmicb.2020.579802)
Supplement: Supplementary Figure 2 — Phylogeny of prophages found in A. baumannii genomes. [file Image_2.pdf]

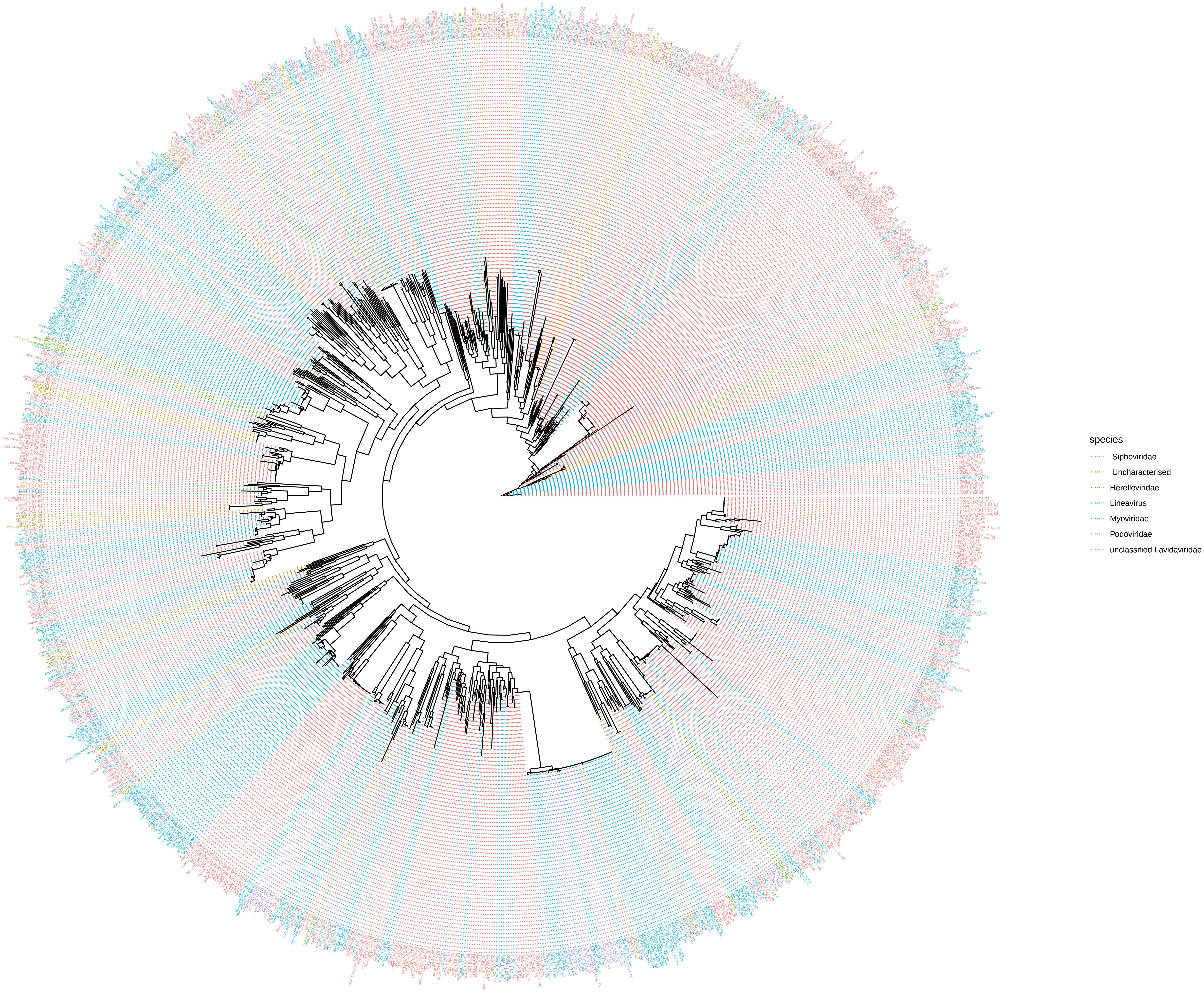
species
Siphoviridae
Uncharacterised
Herelleviridae
Lineavirus
Myoviridae
Podoviridae
unclassified Lavidaviridae
